# Clinical Correlates Identify ProBDNF and Thrombo-Inflammatory Markers as Key Predictors of Circulating p75^NTR^ Extracellular Domain Levels in Older Adults

**DOI:** 10.3389/fnagi.2022.821865

**Published:** 2022-02-21

**Authors:** Samuel Fleury, Mireille E. Schnitzer, Lawrence Ledoux-Hutchinson, Imane Boukhatem, Jean-Christophe Bélanger, Mélanie Welman, David Busseuil, Jean-Claude Tardif, Bianca D’Antono, Marie Lordkipanidzé

**Affiliations:** ^1^Research Centre, Montreal Heart Institute, Montreal, QC, Canada; ^2^Faculty of Pharmacy, Université de Montréal, Montreal, QC, Canada; ^3^Department of Social and Preventive Medicine, School of Public Health, Université de Montréal, Montreal, QC, Canada; ^4^Faculty of Medicine, Université de Montréal, Montreal, QC, Canada; ^5^Department of Medicine, Montreal Heart Institute, Montreal, QC, Canada; ^6^Department of Psychology, Faculty of Arts and Sciences, Université de Montréal, Montreal, QC, Canada

**Keywords:** p75^NTR^ receptor, plasma, proBDNF, neurotrophhic factors, inflammation

## Abstract

The p75^NTR^ receptor binds all neurotrophins and is mostly known for its role in neuronal survival and apoptosis. Recently, the extracellular domain (ECD) of p75^NTR^ has been reported in plasma, its levels being dysregulated in numerous neurological diseases. However, the factors associated with p75^NTR^ ECD levels remain unknown. We investigated clinical correlates of plasma p75^NTR^ ECD levels in older adults without clinically manifested neurological disorders. Circulating p75^NTR^ levels were measured by enzyme-linked immunosorbent assay in plasma obtained from participants in the BEL-AGE cohort (*n* = 1,280). Determinants of plasma p75^NTR^ ECD levels were explored using linear and non-linear statistical models. Plasma p75^NTR^ ECD levels were higher in male participants; were positively correlated with circulating concentrations of pro-brain-derived neurotrophic factor, and inflammatory markers interleukin-6 and CD40 Ligand; and were negatively correlated with the platelet activation marker P-selectin. While most individuals had p75^NTR^ levels ranging from 43 to 358 pg/ml, high p75^NTR^ levels reaching up to 9,000 pg/ml were detectable in a subgroup representing 15% of the individuals studied. In this cohort of older adults without clinically manifested neurological disorders, there was no association between plasma p75^NTR^ ECD levels and cognitive performance, as assessed by the Montreal Cognitive Assessment score. The physiological relevance of high p75^NTR^ ECD levels in plasma warrants further investigation. Further research assessing the source of circulating p75^NTR^ is needed for a deeper understanding of the direction of effect, and to investigate whether high p75^NTR^ ECD levels are predictive biomarkers or consequences of neuropathology.

## Introduction

The brain-derived neurotrophic factor (BDNF) is a protein from the neurotrophin family that is first synthesized as a proprotein, proBDNF, which is cleaved to produce mature BDNF (Lu et al., 2005; Lessmann and Brigadski, 2009). Whereas BDNF is involved in synaptic plasticity by promoting neuronal growth and survival through the binding of the tropomyosin receptor kinase B (TrkB) (Lu et al., 2005), proBDNF instead instigates neuronal apoptosis through its action on the pan-neurotrophic receptor p75^NTR^ (Bothwell, 1991; Nykjaer et al., 2005; Teng et al., 2005; Longo and Massa, 2013). Expression of proBDNF and BDNF is not limited to the central nervous system (Barde et al., 1982), however, as they are both found in higher concentrations in blood. While proBDNF is mostly found in plasma, BDNF is stored in platelets and secreted upon platelet activation (Rosenfeld et al., 1995; Pliego-Rivero et al., 1997; Fujimura et al., 2002; ATLAS Collaboration, 2014; Le Blanc et al., 2020). The p75^NTR^ receptor is mainly expressed during brain development, where it is involved in axonal pruning, but expressed only scarcely in later stages of life. However, p75^NTR^ expression can be induced during adulthood in conditions involving damage to neurons, where it is believed to be involved in neuronal apoptosis of injured cells. The p75^NTR^ receptor is also expressed outside the central nervous system and found on peripheral blood mononuclear cells, on endothelial cells where its expression is increased by ischemia, and on platelets (Salis et al., 2004; Brunelli et al., 2012; Minnone et al., 2017; Fleury et al., 2021). Upon activation, the p75^NTR^ receptor undergoes shedding, causing its extracellular domain (ECD) to be released into circulation (Bergmeier et al., 2004; Weskamp et al., 2004; Duerschmied et al., 2009; Skeldal et al., 2011). Interestingly, elevated circulating p75^NTR^ ECD levels are observed in Alzheimer’s disease and major depressive disorders (Zhou et al., 2013; Jiao et al., 2015), while high urinary p75^NTR^ levels are reported in amyotrophic lateral sclerosis (ALS) and Huntington’s disease patients (Shepheard et al., 2014, 2017; Jia et al., 2017; Simmons et al., 2021). Conversely, reduced circulating levels are reported in schizophrenia, and bipolar mania (Zakharyan et al., 2014; Chen et al., 2017). Circulating p75^NTR^ ECD levels thus have the potential to help diagnose several neurological diseases (Chilton et al., 2004; Jiao et al., 2015; Chen et al., 2017; Shi et al., 2021). However, the factors associated with p75^NTR^ ECD levels in blood remain largely unknown. In this study, we sought to investigate the determinants of circulating p75^NTR^ levels in a large cohort of older adults.

## Methods

### Participant selection

Between September 2012 and June 2017, 1,280 older men and women [*n* = 673 with coronary artery disease (CAD) and *n* = 607 without CAD], were recruited from the André and France Desmarais Hospital Cohort of the Montreal Heart Institute into the BEL-AGE study that seeks to examine the role of psychological burden on pathological aging, as previously described (Gentile et al., 2019; Belanger et al., 2021; Vaillancourt et al., 2021). To do so, the BEL-AGE study follows these 1,280 individuals in time and records information regarding aging and psychological condition, and screens for mild cognitive dysfunction. Participants, termed in this manuscript as older adults, had to be part of the André and France Desmarais Hospital Cohort of the Montreal Heart Institute for at least 5 years, and be between ages 30 and 70 at age of enrollment. The qualification of “older” refers to the age of the final sample, 90.6% of whom fall above the 55-year-old range. While we did not seek specifically to recruit older adults, as indicated by the inclusion criteria, the fact that participants are recruited from a tertiary care facility, half of whom with CAD, necessarily led to recruitment of mostly middle-aged to older individuals. Participants with self-reported or medically documented significant cognitive impairment or psychiatric disorders were excluded from the BEL-AGE cohort. Participants suffering from life-threatening diseases, such as cancer (except for skin cancer) and ALS were also excluded from the cohort. Participants with disorders that did not affect their ability to provide informed consent, such as anxiety and depressive disorders, were not excluded (Vaillancourt et al., 2021). The criteria defining the CAD and non-CAD status were described earlier (Belanger et al., 2021). The CAD status was self-reported and confirmed by medical record review. Serious psychiatric illnesses and cognitive impairment were self-reported, and the latter was further confirmed by the Montreal Cognitive Assessment (MoCA) score. This study was reviewed and approved by the Montreal Heart Institute’s Research Ethics Committee [2011-202 (11-1313)] and all participants gave informed written consent.

### Cognition Assessment

The cognitive status was evaluated using the MoCA score. The MoCA score was developed to detect mild cognitive impairments (Nasreddine et al., 2005). It is a validated test for the assessment of overall cognitive function and sensitive to the early cognitive decline associated with vascular diseases (Hachinski et al., 2006). The test was administered to each participant of the BEL-AGE cohort by a qualified research assistant. Scores were corrected for the education level (one point was added to the score of participants having less than 12 years of education) and the MoCA scores were treated as a continuous variable for analysis.

### Blood Collection

Participants were scheduled for a laboratory appointment between 8:00 and 10:00 AM on a weekday to control for circadian rhythms. They were asked to abstain from eating, drinking (with the exception of water), smoking, and strenuous exercise for 12 h prior to testing. They were also asked to refrain from using illicit drugs or alcohol 24 h preceding their appointment but could continue taking medications as prescribed. Blood was drawn into EDTA-containing collection tubes (BD Vacutainer) and processed within 30 min of collection. The whole blood samples were centrifuged at 4°C at 1,500*g* for 15 min to obtain plasma. Plasma samples were aliquoted at 500 μl per tube and kept frozen at −80°C until analysis. Aliquots were used only once, i.e., with a single freeze-thaw cycle, to avoid protein degradation. Thawed plasma samples were centrifuged at 4°C at 3,000*g* for 5 min to remove debris prior to quantification.

### Quantification of Soluble Factors

Plasma p75^NTR^ ECD and proBDNF levels were assessed by enzyme-linked immunosorbent assay kits (R&D Systems, cat. DY367 and DY3175). Plates (96-well) were coated overnight with the capture antibody at room temperature according to the manufacturer’s recommendations. The next morning, plates were washed thrice with wash solution, blocked in reagent diluent for 60 min and samples were added to the wells for 2 h. Plates were washed thrice and biotinylated detection antibody was added at the concentration recommended by the manufacturer for 2 h. Following three washes, HRP-coupled streptavidin was added and incubated 20 min in the dark. Substrate solution was added for 20 min in the dark, and then stopped by the addition of 2N H_2_SO_4_. Absorbance was read at 450 nm with a 620 nm reference using the Infinite F50 plate reader (Tecan, Männedorf, Switzerland). Each sample was measured in duplicate and the mean was used as representative value. Samples with concentrations exceeding the higher limit of quantification were diluted to reach quantifiable levels and re-measured.

Brain-derived neurotrophic factor, CD40 ligand (CD40L), and soluble P-selectin were quantified by multiplex flow cytometry using a custom kit from Aimplex Biosciences (Pomona, CA, United States), according to the manufacturer’s instructions. Briefly, samples were incubated with beads of different sizes targeting each of the specified analytes and fluorescent secondary antibodies were added. Data was acquired on a MACSQuant Analyzer 10 flow cytometer equipped with the MACSQuantify software (Miltenyi Biotec, Bergisch Gladbach, Germany), and analyzed using the FCAP array software (BD Biosciences, San Jose, CA, United States). Samples were analyzed in duplicate, and the mean was used as representative for each sample.

### Statistical Analysis

Data normality was assessed with the Shapiro–Wilk test. Comparisons between CAD and non-CAD groups or low p75^NTR^ and high p75^NTR^ groups were done using the *t*-test or the Mann–Whitney U test according to the distribution, or with the Chi-square test for dichotomous variables. Univariate linear models were done using IBM SPSS Statistics 25. Multivariate linear models including 23 clinical, biochemical and demographic variables, the adaptive least absolute shrinkage and selection operator (ALASSO) (Zou, 2006; Friedman et al., 2010), and random forests (Liaw and Wiener, 2002) were implemented using R version 4.1.0 (R Development Core Team, 2021). For ALASSO compatibility purposes, skewed continuous covariates were log-transformed then standardized. The 95% post-selection confidence intervals were constructed for the estimated covariate coefficients selected by ALASSO (Tibshirani et al., 2019). For random forests, the number of candidate variables, the maximum number of nodes per tree and the number of trees were tuned by selecting the lowest mean-squared error using 10-fold cross validation. Adjusted *p*-values < 0.05 were considered statistically significant. Graphical representations were plotted using GraphPad Prism Software version 8 for Windows (San Diego, CA, United States).

## Results

### Circulating p75^NTR^ Extracellular Domain Levels Are Highly Variable

**FIGURE 1:**
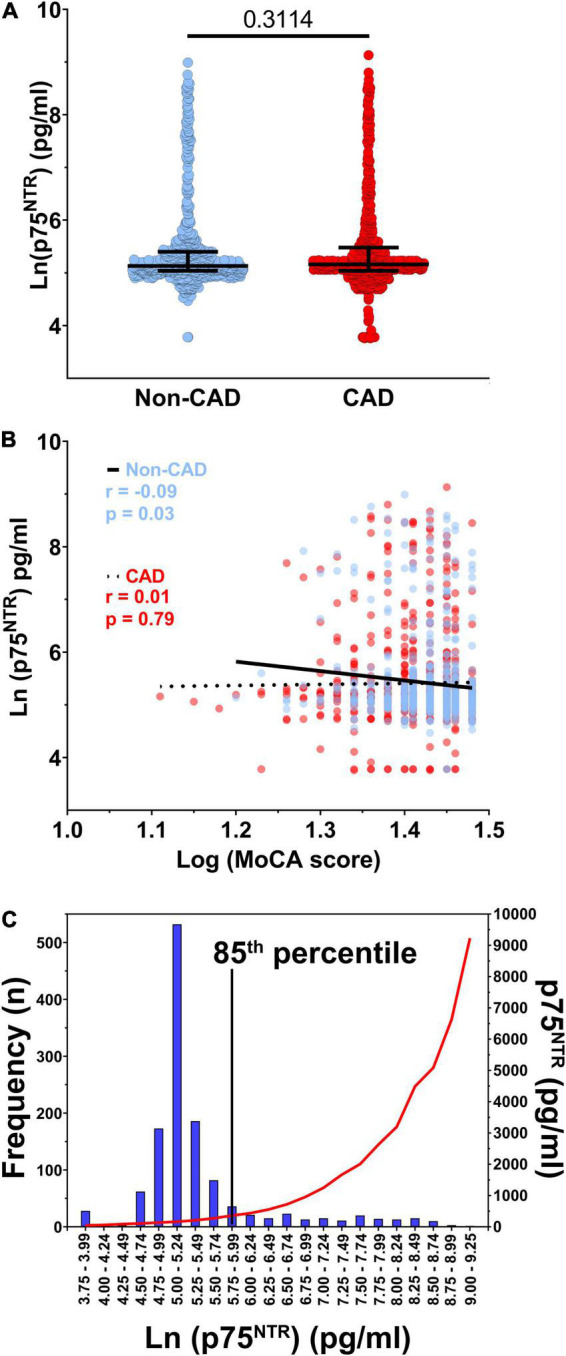
p75^NTR^ distribution and correlation with coronary artery disease. **(A)** Soluble p75^NTR^ extracellular domain (ECD) levels in participants without (blue, *n* = 607) and with coronary artery disease (CAD, red, *n* = 673). **(B)** Spearman correlation between The Montreal Cognitive Assessment (MoCA) score and plasma p75^NTR^ ECD levels in non-CAD (blue, *n* = 607) and CAD (red, *n* = 673) participants. **(C)** Distribution of ln-transformed plasma p75^NTR^ ECD levels (bars, left *y* axis) as well as the non-transformed median p75^NTR^ plasma levels (red curve, right *y* axis), *n* = 1,280. The 85th percentile was chosen as cut-off because levels started to rise exponentially around this percentile.

Circulating p75^NTR^ levels varied between 40 and 9,207 pg/ml and there was no difference in p75^NTR^ ECD levels between CAD and non-CAD individuals (Figure 1A). In this cohort of older adults without clinically manifested neurological disorders, there was no meaningful association between circulating p75^NTR^ levels and cognition, as assessed by the MoCA score (Figure 1B). Based on the graphical distribution presented in Figure 1C and Supplementary Figure 1, participants were divided into two groups according to their plasma p75^NTR^ levels, which were categorized as low (<360 pg/ml) or high (>360 pg/ml). This arbitrary cut-off value corresponds to the 85th percentile and was determined graphically as the levels of p75^NTR^ began to grow exponentially around this percentile (Supplementary Figure 1). We found that most participants had low (40 – 359 pg/ml) p75^NTR^ ECD levels, while the remaining 15% had high (362 – 9,207 pg/ml) p75^NTR^ ECD levels (Figure 1C, Supplementary Figure 1, and Supplementary Table 1). Participants with high p75^NTR^ levels were more frequently male, non-smokers, and had lower HDL cholesterol levels and higher IL-6, CD40L, and proBDNF levels (Table 1), although only proBDNF and IL-6 levels met the Bonferroni-corrected level of significance. We conducted sensitivity analyses using quartiles of p75^NTR^ levels (Supplementary Table 2), which also identified proBDNF and thrombo-inflammatory markers (IL-6, IL-6Rα, CD40L, and P-selectin) as significantly associated with p75^NTR^ levels.

**TABLE 1:** Distribution of baseline characteristics in low and high p75^NTR^ extracellular domain (ECD) groups.

Characteristics	Low p75^NTR^ (*n* = 1,088)	High p75^NTR^ (*n* = 192)	*p*-value	Total (*n* = 1,280)
**Demographics and Lifestyle**				
Age, years *	66.7 (61.7; 70.6)	66.5 (60.7; 70.9)	0.82	66.7 (61.4; 70.7)
Women, *n* (%)	448 (41.2%)	62 (32.3%)	**0.02**	510 (39.8%)
Education, years	14.0 (5.0)	14.0 (5.0)	0.89	14.0 (5.0)
Physical exercise, hours per week *	2.5 (0.0; 4.5)	2.0 (0.0; 4.5)	0.11	2.3 (0.0; 4.5)
Tobacco use, *n* (%)	120 (11.0%)	10 (5.2%)	**0.01**	130 (10.2%)
**Clinical characteristics**				
BMI, kg/m^2^ *	28.6 (25.7; 32.3)	29.0 (25.6; 32.1)	0.99	28.7 (25.7; 32.3)
Hypertension, *n* (%)	581 (53.4%)	116 (60.4%)	0.07	697 (54.5%)
Diabetes, *n* (%)	196 (18.0%)	35 (18.2%)	0.94	231 (18.0%)
Dyslipidemia, *n* (%)	768 (70.6%)	138 (71.9%)	0.72	906 (70.8)
Arrhythmia, *n* (%)	132 (12.1%)	30 (15.6%)	0.18	162 (12.7%)
Coronary artery disease, *n* (%)	561 (51.6%)	112 (58.3%)	0.08	673 (52.6%)
10 year cardiovascular risk,% *	17.8 (10.5; 27.6)	20.1 (12.6; 29.2)	0.06	18.1 (10.8; 27.8)
Montreal Cognitive Assessment, score *	27 (25; 28)	27 (24; 28)	0.16	27 (25; 28)
No impairment, *n* (%)	598 (55.0%)	100 (52.1%)		698 (54.5%)
Mild cognitive impairment, *n* (%)	481 (44.2%)	92 (47.9%)		573 (44.8%)
Moderate cognitive impairment, *n* (%)	9 (0.8%)	0 (0.0%)		9 (0.7%)
**History of vascular events**				
Myocardial infarction, *n* (%)	356 (32.7%)	72 (37.5%)	0.20	428 (33.4%)
Coronary artery graft bypass, *n* (%)	200 (18.4%)	46 (24.0%)	0.07	246 (19.2%)
Percutaneous coronary intervention, *n* (%)	407 (37.4%)	65 (33.9%)	0.35	472 (36.9%)
Stroke, *n* (%)	40 (3.7%)	11 (5.7%)	0.18	51 (4.0%)
**Medication**				
Antiplatelets, *n* (%)	615 (56.5%)	117 (60.9%)	0.17	732 (57.2%)
Anticoagulants, *n* (%)	85 (7.8%)	11 (5.7%)	0.31	96 (7.5%)
Antihypertensive, *n* (%)	709 (65.2%)	136 (70.8%)	0.13	845 (66.0%)
Cholesterol-lowering medications, *n* (%)	741 (68.1%)	138 (71.9%)	0.30	879 (68.7%)
Antidepressants, *n* (%)	122 (11.2%)	25 (13.0%)	0.47	147 (11.5%)
**Laboratory data**				
Systolic blood pressure, mmHg	140.0 (26.8)	143.9 (28.2)	0.61	140.5 (27.1)
Total cholesterol (mmol/L) *	4.1 (3.3; 5.0)	3.8 (3.2; 5.0)	0.11	4.0 (3.3; 5.0)
HDL cholesterol (mmol/L) *	1.3 (1.0; 1.6)	1.2 (1.0; 1.5)	**0.01**	1.3 (1.0; 1.6)
LDL cholesterol (mmol/L) *	2.4 (1.8; 3.2)	2.2 (1.7; 3.2)	0.42	2.3 (1.8; 3.2)
Triglyceride (mmol/L) *	1.5 (1.1; 2.0)	1.5 (1.1; 2.0)	0.78	1.5 (1.1; 2.0)
Glucose (mmol/L) *	5.8 (5.4; 6.4)	5.8 (5.3; 6.6)	0.95	5.8 (5.4; 6.4)
Insulin (pmol/L) *	65 (41; 102)	72 (46; 113)	0.05	66 (41; 104)
C-reactive protein (mg/L) *	1.3 (0.6; 2.8)	1.3 (0.6; 3.0)	0.52	1.3 (0.6; 2.9)
IL-6 (pg/mL) *	1.7 (1.2; 2.6)	2.1 (1.3; 3.7)	<0.01**	1.8(1.2; 2.7)
IL-6Rα (ng/mL)	24,575 (10; 310)	24 892 (10; 116)	0.86	24 627 (10; 187)
P-selectin (pg/ml) *	7,688 (4,380; 16,768)	8,057 (4,662; 16,839)	0.47	7,749 (4,409; 16,809)
CD40L (pg/ml) *	83 (62; 112)	89 (62; 121)	**0.04**	84 (62; 113)
**Neurotrophic factors**				
BDNF (pg/ml) *	810 (462; 1,349)	860 (479; 1,535)	0.29	816 (466; 1,359)
proBDNF (pg/ml) *	1,759 (854; 3,710)	11,474 (4,269; 21,928)	<0.01**	2,047 (976; 5,178)

Baseline characteristics were compared between low p75^NTR^ (n = 1,088) and high p75^NTR^ (n = 192). 10-year cardiovascular risk was calculated using the Framingham Risk Score. Scored out of a possible total of 30 points, severe, moderate, mild, and no cognitive impairment, respectively reflect MoCA scores <10, between 10 and 17, between 18–26 and >26. Continuous variables are expressed as mean (standard deviation) or median (1st quartile; 3rd quartile) when distribution was skewed (identified *). Categorical variables are expressed as n (% of group). For continuous variables, difference between groups was assessed with the t-test or Mann–Whitney U test depending on the normality of the distribution determined using the Kolmogorov–Smirnov test. Chi-square test was used to assess differences in distribution between non-continuous variables. The p-values represent uncorrected difference between low p75^NTR^ and high p75^NTR^ groups. p-values <0.05 are presented in bold. **p-value significant at the corrected threshold of p = 0.00139.

### Key Determinants of Plasma p75^NTR^ Levels Include ProBDNF and Inflammatory Markers

Univariate analyses using ln-transformed p75^NTR^ ECD levels as a continuous variable identified p75^NTR^ ligand proBDNF, thrombo-inflammatory molecules IL-6 and CD40L, and male sex as positively associated with the p75^NTR^ ECD levels in plasma (Table 2). In multivariate linear regression, proBDNF, IL-6, CD40L, male sex, age, P-selectin (a marker of platelet activity), and glucose levels were significantly associated with p75^NTR^ (Table 2). Overall, this multivariate model explained 35.5% of p75^NTR^ ECD level variation (*p* < 0.0001).

**TABLE 2:** Determinants of plasma p75^NTR^ levels.

Model	Univariate linear		Multivariate linear		ALASSO		Random forest	
**Adjusted R-squared**	**–**		**0.3553**		**0.3552**		**–**	
		
**Variable**	**β**	**95%CI**	**β**	**95%CI**	**β**	**95%CI**	**Order of importance**	**% increase in mean squared error of predictions**

ProBDNF	0.55	0.50, 0.59	0.58	0.53, 0.63	0.57	0.53, 0.62	1	106.4
P-selectin	–0.02	–0.08,0.04	–0.12	–0.18, –0.06	–0.12	–0.18, –0.08	2	10.8
BDNF	0.04	–0.02,0.09	0.02	–0.08,0.05	–		3	7
IL-6	0.12	0.06, 0.17	0.06	0.003, 0.11	0.05	0.00, 0.18	4	4.4
CD40L	0.12	0.06, 0.17	0.17	0.12, 0.22	0.16	0.12, 0.22	5	4.2
Sex (male)	0.07	0.03, 0.26	0.22	0.10, 0.34	0.18	0.08, 0.62		1.3
Age	0.4	–0.17,0.96	0.06	0.01, 0.11	0.04	–0.01,0.10		1.8
Glucose	0.01	–0.05,0.07	–0.07	–0.13, –0.003	0.01	–0.09,0.16		3.3
Current smoker	0.08	–0.45,–0.07	0.16	–0.32,0.0003	0.06	–0.31,0.25		1.3
Hypertension	0.05	–0.01,0.21	0.1	–0.004,0.20	0.05	–0.12,0.22		0.8
Coronary artery disease	0.02	–0.07,0.15	0.1	–0.02,0.22	0.02	–0.39,0.21		0.2
HDL cholesterol	0.04	–0.10,0.01	0.05	–0.11,0.02	0.004	–0.07,0.66		0.8
IL-6Raα	0.03	–0.03,0.09	0.04	–0.004,0.06	0.02	–0.08,0.09		0.2
Education years	0.03	–0.03,0.08	0.03	–0.02,0.07	–			0.4
Physical activity	0.02	–0.17,0.08	0.01	–0.12,0.09	–			0.7
Waist circumference	0.04	–0.02,0.07	0.03	–0.10,0.03	–			2.3
Diabetes	0.02	–0.12,0.21	0.15	–0.02,0.31	–			0.2
Dyslipidemia	0.01	–0.14,0.10	0.1	–0.22,0.03	–			0.3
LDL cholesterol	0	–0.06,0.05	0.04	–0.02,0.10	–			0.5
Triglycerides	0.01	–0.07,0.04	0.03	–0.08,0.03	–			1.5
Insulin	0.05	–0.01,0.10	0.02	–0.04,0.09	–			0.5
C-reactive protein	0.03	–0.02,0.09	0.02	–0.04,0.07	–			0.3
Antidepressants	0.03	–0.10,0.25	0.08	–0.06,0.22	–			0.2

*Linear-based univariate, multivariate, and adaptive least absolute shrinkage and selection operator (ALASSO) models, and non-parametric random forests were used to assess the variables associated with p75^NTR^ in the BEL-AGE cohort (63 individuals removed due to missing values, n = 1,217). The 95% confidence intervals are presented for the univariate and multivariate linear models and 95% post-selection confidence intervals are given for the ALASSO coefficient estimates of the selected covariates. Covariates significantly associated with p75^NTR^ are highlighted in green (positive association) and red (negative association) for each linear model. For the random forest model, variable importance is given as % increase in mean squared error of predictions when the variable is randomly permuted; the top five are highlighted in pale yellow. For linear models, skewed continuous variables were log-transformed and standardized for ALASSO compatibility purposes. MSE, mean squared error. β represents standardized coefficients for continuous variables and non-standardized coefficients for binary variables.*

**FIGURE 2:**
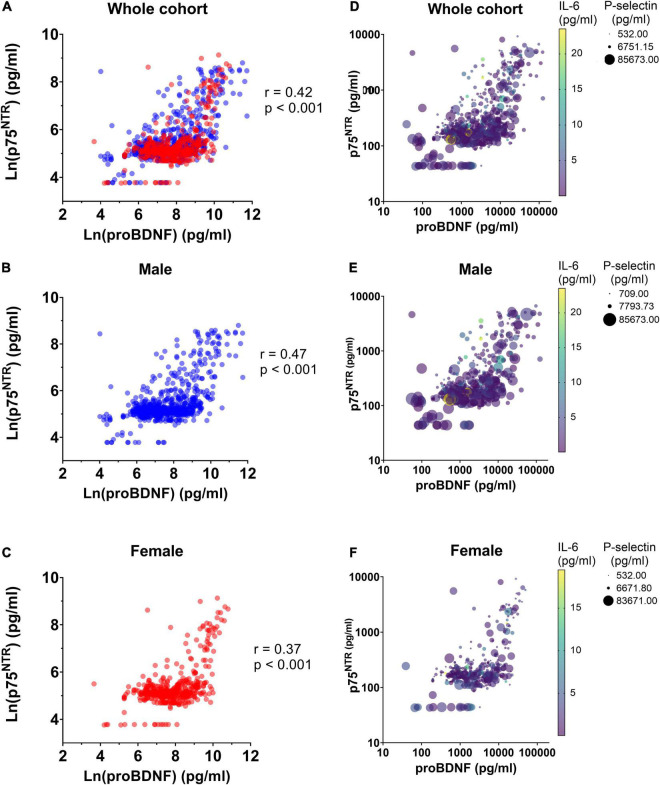
Correlation between p75^NTR^ and proBDNF levels in BEL-AGE participants. Correlation between ln-transformed levels of plasma p75^NTR^ ECD and proBDNF was assessed using Spearman correlation in panel **(A)** the whole BEL-AGE cohort (*n* = 1,280), **(B)** in male participants (*n* = 770) and **(C)** in female participants (*n* = 510). Spearman’s coefficient and *p*-values are displayed. Difference between Spearman’s coefficients in male and female individuals is not significant. Interplay between p75^NTR^, proBDNF, P-selectin, and IL-6 levels in plasma, in panel **(D)** the whole cohort (*n* = 1,280), **(E)** male participants (*n* = 770) and **(F)** female participants (*n* = 510).

We used ALASSO and post-selection inference to reduce the rate of false positive associations. ALASSO identified plasma p75^NTR^ as positively associated with proBDNF, IL-6, CD40L, and male sex, but negatively correlated with P-selectin (Table 2). ProBDNF was identified as the strongest covariate associated to p75^NTR^ ECD levels. Spearman correlations found significant correlation between p75^NTR^ and proBDNF levels in the whole cohort (Figure 2A), as well as in male and female participants separately (Figures 2B,C). Inclusion of IL-6, CD40L and P-selectin did not alter significantly the dominant relationship between proBDNF and p75^NTR^ ECD levels. These covariates are presented graphically in Figure 2D, with the dominant relationship between p75^NTR^ and proBDNF levels being similar in male and female participants (Figures 2E,F).

To explore whether p75^NTR^ levels could be better predicted using non-linear associations, we used the random forest model which identified proBDNF as the strongest determinant of p75^NTR^, followed by P-selectin, BDNF, IL-6, and CD40L (Table 2). In comparison with linear models, the non-parametric random forest model improved the predictive performance over the linear model (measured by cross-validated mean squared error) by 21%, indicating that it was a better fit for p75^NTR^ values.

## Discussion

The results of this study show that in the BEL-AGE cohort of older adults: (1) the plasma concentration of p75^NTR^ ECD is highly variable, with approximately 15% of participants displaying significantly higher circulating levels; (2) the strongest determinant of circulating p75^NTR^ ECD levels were circulating proBDNF levels; and (3) IL-6, CD40L, P-selectin, and male sex were additional predictors of circulating p75^NTR^ ECD levels.

### Origins of Circulating p75^NTR^ Extracellular Domain

The origin of circulating p75^NTR^ ECD remains unknown. Spuch and colleagues identified p75^NTR^ in the choroid plexus epithelium, where it is involved in the transport of amyloid β from cerebrospinal fluid (CSF) to blood (Spuch and Carro, 2011). Amyloid β also induced cleavage of p75^NTR^ and apoptosis of rat primary cell culture of choroid plexus epithelial cells through p75^NTR^ (Spuch and Carro, 2011). Whether the choroid plexus contributes to circulating p75^NTR^ remains elusive and deserves further investigation. Interestingly, Jiao et al. (2015) found that patients suffering from Alzheimer’s disease had lower p75^NTR^ ECD levels in their CSF but high levels in their serum, and that serum levels correlated with cognitive decline. In addition to Alzheimer’s disease, high p75^NTR^ ECD levels have also been found in the urine of patients suffering from ALS, an incurable motor neuron disease. Interestingly, a rise in urinary p75^NTR^ levels was observed before the onset of ALS symptoms in a mouse model (Shepheard et al., 2014). A rise in urinary p75^NTR^ was similarly shown to correlate with disease progression in humans (Shepheard et al., 2014, 2017; Jia et al., 2017). The p75^NTR^ receptor was found to be expressed in motor neurons (Lowry et al., 2001) and Schwann cells (Kerkhoff et al., 1991) of damaged nerves of ALS patients post-mortem, but not in that of healthy age- and sex-matched controls (Kerkhoff et al., 1991), further supporting a pathophysiological role for p75^NTR^. While p75^NTR^ is considered an emergent biomarker for both Alzheimer’s disease and ALS, whether elevated p75^NTR^ levels is predictive of such diseases in humans remains unknown. The long-term follow-up of the cognitive status of the BEL-AGE cohort individuals, who were all free of clinically manifested neurological disorders at enrollment, might help elucidate this question in the near future.

The p75^NTR^ receptor has also been studied in diabetes. Notably, higher plasma concentrations of the full-length receptor were reported in streptozotocin-induced diabetic mice, an effect which disappeared in mice treated with fiderastat, a molecule used to treat diabetic neuropathy, suggesting that the increase in full length p75^NTR^ levels reflected diabetic neuropathic complications rather than diabetes itself (Chilton et al., 2004). Moreover, the p75^NTR^ C-terminal fragment (formed of the transmembrane and intracellular domains) and the cleaved intracellular fragment were reported to be higher in proliferative diabetic retinopathy patients compared to healthy controls (Mysona et al., 2015). None of our models identified diabetes as a plasma p75^NTR^ ECD correlate (Table 2), though we can not exclude that adequate glycemic control in the diabetic participants of the BEL-AGE study might have masked this association, which may only be apparent once diabetic neuropathy is present.

In addition to the nervous system, p75^NTR^ is also expressed in the vascular system. Notably, p75^NTR^ is weakly expressed by endothelial cells, where its expression can be induced under ischemic conditions (Caporali et al., 2008), as well as in peripheral blood mononuclear cells (Fauchais et al., 2008; Brunelli et al., 2012) and platelets (Fleury et al., 2021). Of interest, our data highlight a weak yet significant negative correlation between p75^NTR^ ECD and platelet activation marker P-selectin (Table 2), suggesting that platelet activation could modulate circulating p75^NTR^ ECD levels, and thus sets the basis for future studies regarding platelets as contributors to plasma p75^NTR^ ECD. The marginally higher proportion of patients with hypertension and CAD, mirrored by numerically increased Framingham Risk Scores, could also indicate a role for ischemic events in the rise of p75^NTR^ in previously neurologically healthy subjects, although this would need to be investigated in specifically designed studies.

### Association Between p75^NTR^ Extracellular Domain and ProBDNF

Interestingly, all of our models have shown a strong association between circulating p75^NTR^ ECD levels and proBDNF levels. While p75^NTR^ is found to be upregulated in neurological diseases, making it a potential biomarker of disease, the cleaved p75^NTR^ ECD has also been suggested as a protective neurological factor. In animal studies, circulating p75^NTR^ ECD has exhibited neuro- and cardio-protective effects (Yao et al., 2015; Fang et al., 2020), potentially through quenching of the pro-apoptotic signal of proBDNF (Yu et al., 2018; Fang et al., 2020). Additionally, Yao et al. (2015) have shown that p75^NTR^ ECD in the CSF has a protective role against Alzheimer’s disease by binding to amyloid β, thus preventing its fibrillation and plaque formation. Moreover, Fang et al. (2020) demonstrated that injection of the recombinant p75^NTR^ ECD fragment limited infarct size in a rat model of ischemia reperfusion injury. Since proBDNF can induce apoptosis of microvascular endothelial cells and pericytes *in vitro* (Yu et al., 2018; Fang et al., 2020), we speculate that p75^NTR^ ECD could act as a scavenger to prevent proBDNF-induced endothelial apoptosis. On the other hand, the correlation between plasma proBDNF and p75^NTR^ ECD does not rule out the possibility that proBDNF and p75^NTR^ upregulation could be indirect. Both proteins could be upregulated by the same stimuli (e.g., pro-inflammatory or proapoptotic conditions) without a positive feedback of p75^NTR^ levels on proBDNF expression and vice-versa. Whether proBDNF is bound to p75^NTR^ ECD in plasma remains an open question, and p75^NTR^ ECD could also be bound to other proteins. For instance, the full-length p75^NTR^ receptor was also shown to be involved in amyloid β-induced apoptosis of cholinergic neurons in mice, where ECD cleavage was found to be increased (Sotthibundhu et al., 2008). As it stands, whether circulating p75^NTR^ ECD is actively neuroprotective or simply indicative of underlying neuronal damage *via* p75^NTR^ signaling remains unknown. Further investigations characterizing the interaction of p75^NTR^ ECD with proBDNF and other plasma components is needed to better understand its role.

### Association Between p75^NTR^ Extracellular Domain and Inflammation

We have found that, after proBDNF, inflammatory factors IL-6 and CD40L were the strongest positive predictors of elevated plasma p75^NTR^. Elevated serum IL-6 and CD40L have been reported in many neurological diseases, including multiple sclerosis, Alzheimer’s disease, Parkinson’s disease, and neuromyelitis optica spectrum disorder (Desideri et al., 2008; Giunta et al., 2010; Rothaug et al., 2016; Du et al., 2020; Fujihara et al., 2020). Elevated IL-6 levels have also been proposed as a potential biomarker for severe course in ALS patients, akin to urinary p75^NTR^ ECD, though this finding has so far only been demonstrated in carriers of the IL6R 358 Ala variant (Casault et al., 2019; Wosiski-Kuhn et al., 2021).

However, since IL-6 and CD40L can be increased in patients who have recently or previously suffered traumatic brain injury and central nervous system infections, neither of which were recorded in our cohort, they may represent a confounding factor in the association between p75^NTR^ ECD and IL-6 and CD40L. Moreover, p75^NTR^ ECD was not correlated with C-reactive protein levels, one of the primary markers of acute inflammation. Since C-reactive protein induction is one of the primary functions of cytokine IL-6, it is difficult to explain why elevated p75^NTR^ should be associated to IL-6, but not one of its immediate downstream products (Bermudez et al., 2002). Likewise, elevated p75^NTR^ ECD was not associated with CAD, which is characterized by a hightened inflammatory state including elevated levels of IL-6 and C-reactive protein (Medina-Leyte et al., 2021). Since C-reactive protein usually functions as an acute phase reactant, it is possible that patients with chronically elevated serum IL-6 levels will have normal C-reactive protein outside of acute stresses (Bermudez et al., 2002). Patients in the CAD group were also commonly receiving statin therapy, which is known to significantly reduce C-reactive protein levels due to its anti-inflammatory properties (Albert et al., 2001). It is therefore unclear if p75^NTR^ ECD plays a direct role in inflammatory diseases. Nonetheless, these findings suggest a link between circulating p75^NTR^ levels and immune modulators such as IL-6 and CD40L. Whether elevated p75^NTR^ levels promote inflammation or are simply a result of an underlying inflammatory response remains uncertain (Choi and Friedman, 2009; Rezaee et al., 2010; Bandola et al., 2017). Further investigation is warranted to better understand the causal relationship between p75^NTR^ ECD and neuroinflammatory processes.

### p75^NTR^ Extracellular Domain as a Biomarker

The present study identified high p75^NTR^ levels in 15% of the BEL-AGE individuals. The p75^NTR^ receptor is recently being proposed as a diagnostic biomarker of Alzheimer’s disease in serum (Jiao et al., 2015), and was also proposed as a prognostic, disease progression and potential pharmacodynamic biomarker for ALS and Huntington’s disease in urine, with increase in p75^NTR^ ECD happening prior to symptoms appearance in ALS (Shepheard et al., 2014, 2017; Shi et al., 2021; Simmons et al., 2021). Whether this predictive pattern is specific to these diseases remains unknown. As elevated circulating p75^NTR^ ECD levels have previously been observed in multiple pathologies, that all include neuronal dysfunction or damage, we argue that p75^NTR^ ECD could rather be a more general marker of neuronal damage. Whether the high levels of plasma p75^NTR^ ECD observed here could be indicative of a yet to be diagnosed pathology remains an open question. One caveat to these findings is that p75^NTR^ ECD was not found to be correlated to any disease state. While this might seem to be contradictory with the current literature, this could be due to the absence of life-threatening diseases (i.e., ALS) and major cognitive deficits (i.e., Alzheimer’s diseases dementia) in the BEL-AGE cohort at the time of enrollment. The absence of link between p75^NTR^ and such diseases could thus be explained by the fact that participants that will eventually be diagnosed with those pathologies are still in the early stages of such diseases, and/or that these diseases are not yet symptomatic/diagnosed in these participants. This would be consistent with p75^NTR^ levels rising prior to the onset of symptoms, at least in ALS (Shepheard et al., 2014). The follow-up of the BEL-AGE cohort in the next years will hopefully help to answer this open question. In addition to the possibility of p75^NTR^ as an early diagnostic biomarker, its role could be extended to disease progression (Shepheard et al., 2017), and also be of interest as a pharmacodynamic biomarker to assess the effect of treatment, which is lacking in multiple neurological and psychiatric diseases (Simmons et al., 2021). These latter possibilities have been extensively discussed by Shepheard et al. (2017) and Simmons et al. (2021) and were also discussed in a recent meta-analysis by Shi et al. (2021).

### Limitations

It is important to note that the cross-sectional design of the study does not allow assessment of causality or direction of effect. The selection of a cohort of older adults with and without CAD, and free of clinically manifested neurological disorders, represents a strength but also limits interpretation into neuropathological levels of p75^NTR^ in plasma. While p75^NTR^ ECD levels did not differ between CAD status in the present study, CAD is overrepresented in this cohort compared to the general population, which may limit the inference on the general population. Furthermore, individuals in the BEL-AGE cohort were free of clinically manifested neurological disorders but could be affected by other diseases affecting the central nervous system, for instance depression or anxiety disorders. Indeed, antidepressant use was higher in the highest p75^NTR^ quartile, although this difference did not reach the Bonferroni-corrected level of significance (Supplementary Table 2). While the use of anti-depressant agents was used as a proxy for some of these diseases, we cannot rule out the presence of either untreated participants, participants treated through other drug classes or participants with diseases that are not treated by anti-depressants, for instance attention deficit disorders. The presence of possible concomitant diseases of neurological, psychiatric, or inflammatory orders should thus be considered as a possible confounding factor. In addition, ethnic differences were not investigated, as the BEL-AGE cohort was mostly comprised of French-speaking Caucasian individuals. The association between circulating p75^NTR^ ECD levels and inflammatory markers thus needs to be replicated in other robustly phenotyped cohorts composed of individuals of different ethnicities and clinical characteristics.

Another point worth discussing is that p75^NTR^ levels were not associated with the MoCA score. MoCA was specifically designed and approved for the detection of vascular cognitive impairment (Hachinski et al., 2006). While one strength of the MoCA is that it can detect multiple types of mild and moderate cognitive impairments, previous reports challenged its specificity, sometimes resulting in healthy individuals being considered cognitively impaired (Coen et al., 2011). We cannot exclude that different results could be obtained using different (and more disease-specific) cognitive tests.

## Conclusion

Our results shed light on factors associated with circulating p75^NTR^ ECD levels in older individuals. Identification of these factors will hopefully contribute to a growing understanding of p75^NTR^ ECD levels and their involvement in neurodegenerative diseases and neuroinflammation.

## Data Availability Statement

The raw data supporting the conclusions of this article will be made available by the authors, without undue reservation.

## Ethics Statement

The studies involving human participants were reviewed and approved by the Research Ethics Committee of the Montreal Heart Institute. The patients/participants provided their written informed consent to participate in this study.

## Author Contributions

SF performed the assays, collected the data, analyzed and interpreted the data, and wrote the first draft of the manuscript. MS performed the statistical modeling and provided the draft text and tables. LL-H interpreted the data and contributed to draft the manuscript. IB, J-CB, and MW performed the assays and collected and interpreted the data. DB has supervised the biobank sample access. J-CT is the chair of the MHI Biobank’s management committee, and contributed to the BEL-AGE study design, and funding and conduct. BD’A designed, led, and obtained funding for the BEL-AGE cohort study, and interpreted the data. ML designed the research, oversaw the research group, assured the funding, and analyzed and interpreted the data. All authors critically revised the manuscript and approved the final version.

## Conflict of Interest

ML has received speaker fees from Bayer; has participated in industry-funded trials from Idorsia; has served on advisory boards for Servier and JAMP/Orimed Pharma; and has received in-kind and financial support for investigator-initiated grants from Leo Pharma, Roche Diagnostics, Aggredyne, and Fujimori Kogyo. MS has received speaker fees from Biogen and consultant fees from Carebook Technologies Inc. J-CT reports research grants from Amarin, AstraZeneca, Ceapro, DalCor Pharmaceuticals, Esperion, Ionis, Novartis, Pfizer, and Sanofi; honoraria from AstraZeneca, DalCor Pharmaceuticals, HLS Pharmaceuticals, and Pendopharm; and minor equity interest in DalCor Pharmaceuticals. The remaining authors declare that the research was conducted in the absence of any commercial or financial relationships that could be construed as a potential conflict of interest.

## Publisher’s Note

All claims expressed in this article are solely those of the authors and do not necessarily represent those of their affiliated organizations, or those of the publisher, the editors and the reviewers. Any product that may be evaluated in this article, or claim that may be made by its manufacturer, is not guaranteed or endorsed by the publisher.
