# Supplementary material for: Clinical Correlates Identify ProBDNF and Thrombo-Inflammatory Markers as Key Predictors of Circulating p75NTR Extracellular Domain Levels in Older Adults
Source: Front Aging Neurosci. 2022 Feb 21;14:821865. doi: 10.3389/fnagi.2022.821865 (PMC8899540; doi:10.3389/fnagi.2022.821865)
Supplement: Supplementary file 1 [file Data_Sheet_1.pdf]

Supplementary Material to

Clinical correlates identify proBDNF and thrombo-inflammatory markers as key predictors of circulating p75^{NTR} extracellular domain levels in older adults

Samuel Fleury[1,2], Mireille E. Schnitzer[2,3], Lawrence Ledoux-Hutchinson[4], Imane Boukhatem[1,2], Jean-Christophe Bélanger[1,2], Mélanie Welman[1], David Busseuil[1], Jean-Claude Tardif[1,4,5], Bianca D'Antono[1,6*], Marie Lordkipanidzé[1,2*]

[1] Research Center, Montreal Heart Institute, Montreal, QC, Canada,

[2] Faculty of Pharmacy, Université de Montréal, Montreal, QC, Canada,

[3] Department of Social and Preventive Medicine, School of Public Health, Université de Montréal

[4] Faculty of Medicine, Université de Montréal, Montreal, QC, Canada

[5] Department of Medicine, Montreal Heart Institute, Montreal, Québec, Canada

[6] Psychology Department, Faculty of Arts and Sciences, Université de Montréal, Montreal, Qc, Canada

[*] These authors have contributed equally to this work and share last authorship.

*** Correspondence:**
Bianca D'Antono or Marie Lordkipanidzé, Research Center, Montreal Heart Institute, 5000 rue Bélanger, Montréal, Québec, Canada H1T 1C8

Tel.: +1 514 376 3330; Fax: +1 514 376 0173

e-mail: bianca.d.antono@umontreal.ca / marie.lordkipanidze@umontreal.ca

1 Supplementary Figure

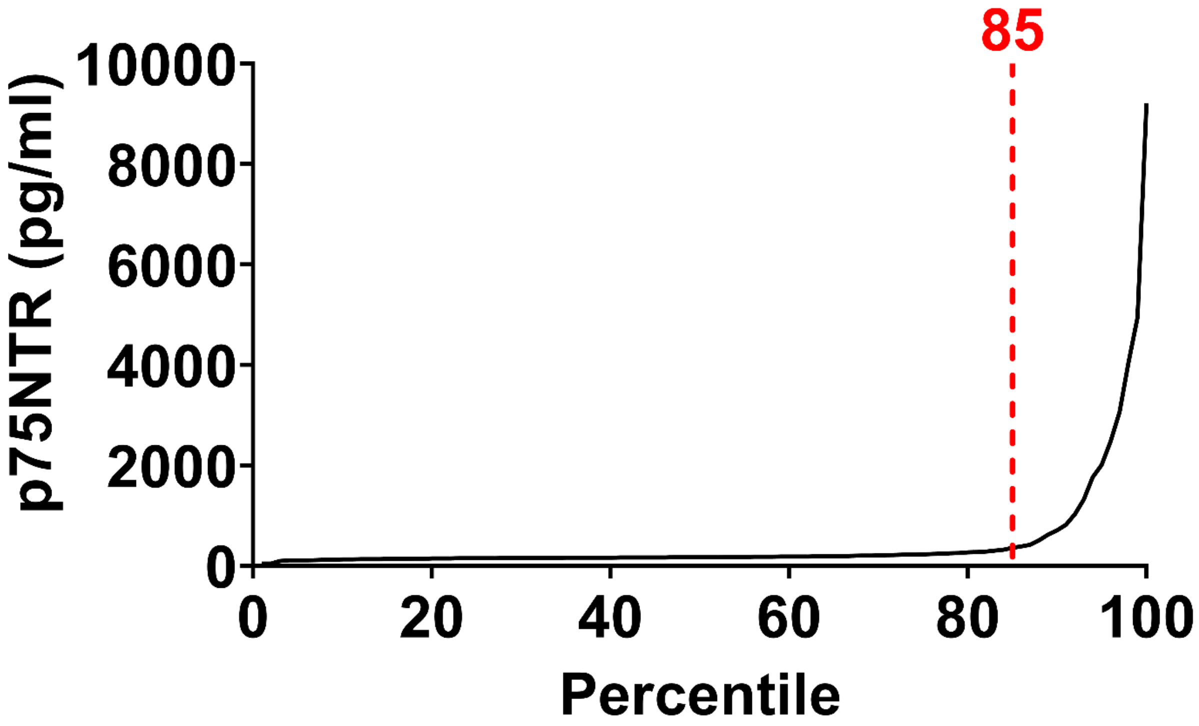

<u>Figure S1. Cutoff determination for low and high p75^{NTR} groups.</u> Plasma p75^{NTR} extracellular domain levels as a function of percentile was used to graphically determine the cut off for low and high p75^{NTR} levels. The 85th percentile was chosen as levels started to raise exponentially around this percentile.

2 Supplementary Tables

	**Low p75^{NTR}**	**High p75^{NTR}**
n (%)	1088 (85)	192 (15)
mean (SD)	173 (50)	1866 (1675)
Minimum	43	362
25th percentile	148	580
Median	166	1239
75th percentile	192	2709
Maximum	358	9207

Table S1. p75^{NTR} levels in p75^{NTR} low and p75^{NTR} high groups. Then BEL-AGE cohort was divided in two groups according to their plasma p75^{NTR} levels being low (<360 pg/ml) or high (>360 pg/ml). The cut-off was determined graphically according to the distribution of p75^{NTR} levels in the BEL-AGE cohort (**Fig 1C**). The distribution of p75^{NTR} levels across both groups is described using extreme values, mean and median as well as 25th and 75th percentiles, all expressed in pg/ml. SD = standard deviation.

Characteristics	1st Quartile (n=320)	2nd Quartile (n=320)	3rd Quartile (n=320)	4th Quartile (n=320)	p-value	Total (n=1280)
p75NTR levels, min - max (pg/ml)	43 .0 - 154.0	154.0 - 172.0	172.0 - 230.0	231.0 - 9207.0	-	43.0 - 9207.0
Demographics and Lifestyle						
Age, years*	66.5 (60.9; 70.4)	66.2 (61.3; 70.4)	67.6 (62.2; 71.5)	66.3 (60.9; 70.7)	0.048	66.7 (61.4; 70.7)
Women, n (%)	143 (44.7%)	136 (42.5%)	119 (37.2%)	112 (35.0%)	0.04	510 (39.8%)
Education, years	14.1 (3.5)	14.6 (3.7)	14.1 (3.7)	14.5 (3.6)	0.32	14.0 (5.0)
Physical exercise, hours per week *	2.5 (0.0; 4.5)	2.5 (0.0; 5.0)	2.5 (0.0; 4.5)	2.0 (0.0; 4.5)	0.048	2.3 (0.0; 4.5)
Smoker, n (%)	37 (11.6%)	36 (11.3%)	35 (11.0%)	22 (6.9%)	0.16	130 (10.2%)
Clinical characteristics						
BMI, kg/m2 *	28.4 (25.5; 31.6)	28.2 (25.4; 32.4)	28.9 (26.2; 32.3)	29.0 (25.8; 32.7)	0.53	28.7 (25.7; 32.3)
Hypertension, n (%)	168 (52.5%)	168 (52.5%)	167 (52.2%)	194 (60.6%)	0.09	697 (54.5%)
Diabetes, n (%)	63 (19.7%)	51 (15.9%)	53 (16.6%)	64 (20.0%)	0.42	231 (18.0%)
Dyslipidemia, n (%)	228 (71.3%)	216 (67.5%)	227 (70.9%)	235 (73.4%)	0.42	906 (70.8%)
Arythmia, n (%)	35 (10.9%)	38 (11.9%)	45 (14.1%)	44 (13.8%)	0.58	162 (12.7%)
Coronary artery disease, n (%)	167 (52.2%)	146 (45.6%)	179 (55.9%)	181 (56.6%)	0.021	673 (52.6%)
10 year cardiovascular risk, % *	17.3 (10.7; 26.9)	17.2 (9.3; 26.9)	18.7 (10.8; 28.0)	18.9 (12.9; 28.7)	0.16	18.1 (10.8; 27.8)
Montreal Cognitive Assessment, score *	27 (25; 28)	27 (25; 28)	27 (24; 28)	27 (25; 28)	0.09	27 (25; 28)
No impairment, n (%)	192 (60.0%)	170 (53.1%)	168 (52.5%)	168 (52.5%)		698 (54.5%)
Mild cognitive impairment, n (%)	126 (39.4%)	148 (46.3%)	148 (46.3%)	151 (47.2%)		573 (44.8%)
Moderate cognitive impairment, n (%)	2 (0.6%)	2 (0.6%)	4 (1.3%)	1 (0.3%)		9 (0.7%)
History of cardiovascular events and interventions						
Myocardial Infarction, n (%)	99 (30.9%)	106 (33.1%)	111 (34.7%)	112 (35.0%)	0.65	428 (33.4%)
Coronary artery bypass graft, n (%)	50 (15.6%)	47 (14.7%)	71 (22.2%)	78 (24.4%)	0.003	246 (19.2%)
Percutaneous coronary intervention, n (%)	126 (39.4%)	109 (34.1%)	127 (39.7%)	110 (34.4%)	0.27	472 (36.9%)
Stroke, n (%)	10 (3.1%)	11 (3.4%)	10 (3.1%)	20 (6.3%)	0.12	51 (4.0%)
Medication						
Antiplatelets, n (%)	190 (59.4%)	163 (50.9%)	184 (57.5%)	195 (60.9%)	0.06	732 (57.2%)
Anticoagulants, n (%)	21 (6.6%)	27 (8.4%)	28 (8.8%)	20 (6.3%)	0.52	96 (7.5%)
Antihypertensive, n (%)	203 (63.4%)	201 (62.8%)	212 (66.3%)	229 (71.6%)	0.08	845 (66.0%)
Cholesterol-lowering medications, n (%)	227 (70.9%)	201 (32.8%)	218 (68.1%)	233 (72.8%)	0.04	879 (68.7%)
Antidepressants, n (%)	25 (7.8%)	39 (12.2%)	34 (10.6%)	49 (15.3%)	0.03	147 (11.5%)
Laboratory data						
Systolic blood pressure, mmHg	141.8 (21.8)	140.1 (22.0)	139.7 (21.5)	141.9 (21.7)	0.45	140.5 (21.7)
Total cholesterol (mmol/L)*	4.0 (3.4; 4.9)	4.1 (3.4; 5.1)	4.0 (3.3; 4.9)	3.9 (3.2; 5.0)	0.15	4.0 (3.3; 5.0)
HDL cholesterol (mmol/L)*	1.3 (1.1; 1.6)	1.3 (1.1; 1.6)	1.3 (1.0; 1.5)	1.2 (1.0; 1.5)	0.01	1.3 (1.0; 1.6)
LDL cholesterol (mmol/L)*	2.3 (1.8; 3.1)	2.4 (1.8; 3.3)	2.4 (1.8; 3.2)	2.3 (1.7; 3.2)	0.48	2.3 (1.8; 3.2)
Triglyceride (mmol/L)*	1.5 (1.1; 2.0)	1.4 (1.1; 2.0)	1.5 (1.1; 2.0)	1.5 (1.1; 2.0)	0.29	1.5 (1.1; 2.0)
Glucose (mmol/L)*	5.8 (5.4; 6.5)	5.8 (5.4; 6.3)	5.8 (5.4; 6.4)	5.8 (5.4; 6.7)	0.63	5.8 (5.4; 6.4)
Insulin (pmol/L)*	63 (41; 101)	63 (38; 102)	67 (44; 99)	71 (44; 117)	0.07	66 (41; 104)
C-reactive protein (mg/L)*	1.1 (0.6; 2.4)	1.2 (0.6; 2.7)	1.4 (0.7; 3.0)	1.3 (0.7; 3.1)	0.11	1.3 (0.6; 2.9)
IL-6 (pg/mL)*	1.6 (1.1; 2.3)	1.7 (1.1; 2.5)	1.9 (1.4; 2.8)	2.0 (1.3; 3.3)	<0.001 (A)	1.8 (1.2; 2.7)
IL-6Ra (ng/mL)	24 299 (7243)	24 815 (7970)	26 660 (7913)	25 588 (8251)	<0.001 (B)	24 627 (10 187)
P-selectin (pg/ml)*	8954 (4805; 30644)	8068 (4305; 16571)	6887 (4351; 12251)	7770 (4352; 15993)	<0.001 (C)	7749 (4409; 16 809)
CD40L (pg/ml)*	73 (61; 96)	85 (60; 113)	85 (64; 119)	89 (65; 119)	<0.001 (D)	84 (32; 113)
Proteins of interest						
BDNF (pg/ml)*	869.0 (521.0; 1344.5)	733.5 (403.5; 13058.5)	826.0 (458.5; 1347.5)	854.5 (471.0; 1562.0)	0.07	816 (466; 1359)
proBDNF (pg/ml)*	1473 (630; 2890)	1767 (651; 3023)	1607 (827; 3581)	7637 (2736; 16157)	<0.001 (E)	2047 (976; 5178)

Table S2. Distribution of baseline characteristics in quartiles based on p75^{NTR} levels. Baseline characteristics were compared between quartiles based on p75^{NTR} levels (n=320 per quartile). Continuous variables are expressed as mean (standard deviation) or median (1st quartile; 3rd quartile) when distribution was skewed (identified *). Categorical variables are expressed as n (% of group). For continuous variables, difference between groups was assessed using an ANOVA or Kruskal-Wallis test depending on the normality of the distribution determined using the Kolmogorov-Smirnov test. Chi-square test was used to assess differences in distribution between non-continuous variables.

The p-values represent uncorrected differences. Letters in parenthesis = p-value significant at the Bonferroni corrected threshold of $p = 0.00139$.

(A) Q1 vs Q3 $p = 0.001$; Q1 vs Q4 $p < 0.001$; Q2 vs Q3 $p = 0.02$; Q2 vs Q4 $p = 0.001$.

(B) Q1 vs Q3 $p = 0.002$; Q2 vs Q3 $p = 0.008$.

(C) Q1 vs Q2 $p=0.02$; Q1 vs Q3 $p < 0.001$; Q1 vs Q4 $p = 0.03$.

(D) Q1 VS Q3 $p = 0.05$; Q1 vs Q4 $p < 0.001$.

(E) Q1 vs Q4 $p < 0.001$; Q2 vs Q4 $p < 0.001$; Q3 vs Q4 $p < 0.001$.

10-year cardiovascular risk was calculated using the Framingham Risk Score. Scored out of a possible total of 30 points, severe, moderate, mild and no cognitive impairment respectively reflect MoCA scores <10, between 10-17, between 18-26 and ≥26.
